# Acute Sinusitis Resulting in a Craniotomy: An Uncommon Complication of a Common Infection

**DOI:** 10.1155/2012/979836

**Published:** 2012-08-16

**Authors:** Allison Price, Arjun Mohan, Larry M. Bush

**Affiliations:** ^1^JFK Medical Center and Division of General Internal Medicine, Miller School of Medicine, University of Miami, Miami, FL 33136, USA; ^2^Department of Biomedical Sciences, Charles E. Schmidt College of Medicine, Florida Atlantic University, Boca Raton, FL 33431, USA; ^3^JFK Medical Center and Department of Medicine, Miller School of Medicine, University of Miami, Miami, FL 33136, USA

## Abstract

Acute bacterial sinusitis is a common infectious condition. Patients may initially present with an uncomplicated infection and later, despite appropriate initial antibiotic therapy, develop a potentially life-threatening complication. Interventions aimed at alleviating such unexpected events need be prompt and adequate. We describe a case of a patient who initially presented with signs and symptoms of acute sinusitis later to be diagnosed with a frontal epidural abscess.

## 1. Introduction

Complications of acute bacterial sinusitis can be serious and may necessitate urgent surgical treatment. The floor of the frontal sinus makes up the roof of the orbit, while the posterior border of the frontal sinus is in close contact with the neighboring dura [1], with this proximity being a likely cause of complications. Due to a relative increase in blood supply to the sinuses, as well as a greater incidence of acute sinusitis, children greater than six years old and adolescents are at greater risk of complications from these pyogenic upper respiratory tract infections [2]. A male predominance has been reported [3–7]. As with our patient, a prior history of sinusitis is commonly elicited in persons who are diagnosed with intracranial or orbital complications [3]. Moreover, it is not unusual for patients to be on antibiotic therapy when the abscess develops [5]. 

## 2. Case Report

An 18-year-old man, with no pertinent past medical history, presented to the emergency department (ED) complaining of clinical features consistent with acute bacterial sinusitis. A computerized tomography scan (CT) of the sinuses was performed, but failed to show acute pathology. The patient was discharged home with oral levofloxacin, and instructed to follow up with a local primary care clinic.

Two days later, the patient returned to the ED with significant left eyelid swelling, headache, and fever. Physical examination was remarkable for left eye gaze palsy, severe eyelid edema, and marked erythema. Laboratory data proved normal, including no leukocytosis. A repeat CT now revealed findings consistent with a left frontal epidural abscess. 

The Infectious Diseases service was consulted and broad-spectrum intravenous (IV) antibiotic therapy (meropenem and vancomycin) was empirically initiated. The following day the patient underwent a left frontal sinus cranialization with bicoronal craniotomy, for sinus and abscess drainage.

Surgical culture revealed methicillin-sensitive *Staphylococcus aureus* and antibiotic therapy was changed to IV oxacillin. His postoperative hospital course was uneventful. Following 2 weeks of in-hospital care he was discharged home with outpatient antibiotic therapy (IV ceftriaxone 2 g every 12 hours) to be continued for an additional three weeks. He recovered fully without evidence of sequelae.

## 3. Discussion

Acute bacterial sinusitis (ABS) should be empirically treated in any of the following clinical scenarios:

1. presence of moderate signs/symptoms for more than 10 days without improvement [8];
2. severe symptoms or fever greater than 30 degrees Celsius with purulent nasal drainage and/or facial pain [8];
3. worsening symptoms with new fever, headache, and/or increased nasal drainage 5-6 days after a URI that initially improved [8].

First-line empiric treatment for uncomplicated ABS is amoxicillin-clavulanate for 5–7 days in adults or 10–14 days in children [8]. Symptoms that worsen 48–72 hours after treatment initiation, or fail to improve after 3–5 days, should be reassessed [8]. 

Intracranial complications of acute bacterial sinusitis often have diverse presenting signs and symptoms, which may include headache, seizures, and meningismus [7]. However, some patients may present void of neurologic signs or symptoms [2]. A CT scan is the recommended initial imaging modality, with MRI scans being reserved as an alternative or adjunct study when there remains a high index of suspicion for an intracranial infectious process in the setting of a negative CT scan [3, 6]. 

Retrospective studies indicate that the etiologic agent of intracranial infections complications associated with acute bacterial sinusitis is typically monomicrobial [4, 5] with *Streptococcal species* being isolated most commonly [2, 4–7]. However, *Staphylococcal species* are the predominant pathogens associated with sinusitis-related epidural abscess formation [7]. Lacking evidence from controlled treatment trials, anti-infective therapy consisting of an advanced generation cephalosporin (ceftriaxone) along with an anti-anaerobic agent (metronidazole), has proven to be quite successful. These are a common treatment of upper respiratory tract related intracranial bacterial infection complications [4–6, 9]. Vancomycin should be included in those instances where methicillin-resistant *S. aureus* is a proven or suspected pathogen [4]. In general, IV antimicrobial therapy is given for four to eight weeks, the majority of which can be administered in the outpatient setting [5, 6].

The incidence of long-term sequelae following combined surgical and antimicrobial therapy is infrequent, having been reported in less than 15% of cases [2–5]. Mortality is rare and usually is attributed to complications of severe infection, such as sepsis and respiratory failure [2]. 

## 4. Conclusion

Intracranial complications of acute bacterial sinusitis are uncommon, but necessitate appropriate evaluation and treatment when clinically suspected. CT scan is the diagnostic imaging modality of choice in the acute setting. Empiric antimicrobial therapy should be selected based upon knowledge of the normal upper respiratory bacterial flora, taking into consideration the risk of multi-drug resistant organisms as well as unusual pathogens if the epidemiologic setting suggests the possibility of their presence. In addition, if indicated, prompt surgical drainage should be performed. Transition to the outpatient setting, to complete the required lengthy IV antimicrobial therapy, is achievable in the majority of cases.
